# Case Report: Vogt-Koyanagi-Harada-like uveitis associated with combined dabrafenib/trametinib and cadonilimab therapy for cutaneous melanoma

**DOI:** 10.3389/fmed.2026.1804008

**Published:** 2026-06-24

**Authors:** Han Wang, Ran Zhang, Li Zhou, Qian Liang, Hong Ma, Xiaomei Nie, Gang Su

**Affiliations:** Department of Ophthalmology, The Second Affiliated Hospital of Zunyi Medical University, Zunyi, Guizhou, China

**Keywords:** drug adverse reactions, BRAF and MEK inhibitors, case report, immune checkpoint inhibitor, melanoma, Vogt-Koyanagi-Harada-like uveitis

## Abstract

**Background:**

Several BRAF kinase inhibitors (BRAFis), MEK kinase inhibitors (MEKis), and immune checkpoint inhibitors have been linked with the occurrence of Vogt-Koyanagi-Harada (VKH)-like uveitis. However, there are no reported cases of VKH-like uveitis induced by the combined administration of dabrafenib, trametinib, and cadonilimab to date.

**Case presentation:**

This paper presents a case of VKH-like uveitis in a patient with inguinal melanoma following combined treatment with dabrafenib, trametinib, and cadonilimab. Four months after the initiation of this combined therapy, the patient developed severe bilateral acute panuveitis complicated by exudative retinal detachment, and a diagnosis of bilateral VKH-like uveitis was considered. The patient’s ocular condition of both eyes improved after the discontinuation of dabrafenib and trametinib, along with the administration of topical and systemic glucocorticoid therapy. At the time of drug discontinuation, the patient’s metastatic liver lesions remained stable, and no melanoma progression was observed during the 1-year follow-up.

**Conclusion:**

This report demonstrates that VKH-like uveitis may occur in patients receiving combined treatment with dabrafenib, trametinib, and cadonilimab, and emphasizes the importance of monitoring for ocular complications in patients undergoing long-term treatment with this regimen.

## 1 Introduction

Vogt-Koyanagi-Harada syndrome is a rare autoimmune disease characterized by bilateral granulomatous uveitis as the core manifestation, often accompanied by meningeal irritation signs, cutaneous and hair changes, and auditory abnormalities (1). Studies have shown that this disease accounts for 37.7% of all panuveitis patients in China,

predominantly affecting adults aged 20–50 years with no significant gender difference (2, 3). Without timely intervention, it can lead to severe complications such as retinal detachment and optic atrophy, ultimately resulting in irreversible visual loss. The known predisposing factors for VKH syndrome include infection, genetic factor, and immune dysregulation; however, drug-induced VKH-like uveitis has been rarely reported in clinical practice, especially in the field of anti-tumor therapy.

The combination of dabrafenib and trametinib (DabTram) is a classic targeted therapy regimen for malignant tumors with V-raf murine sarcoma viral oncogene homologue B1 (BRAF) V600 mutation. By inhibiting the mitogen-activated protein kinase (MAPK) signaling pathway to block the proliferation and survival of tumor cells, it has been widely used in the treatment of malignant tumors such as melanoma and non-small cell lung cancer (4, 5). Cadonilimab is a programmed cell death protein-1 (PD-1)/cytotoxic T-lymphocyte associated antigen-4 (CTLA-4) bispecific antibody that enhances anti-tumor immune responses by simultaneously blocking two immune checkpoint pathways, providing a new therapeutic option for patients with advanced solid tumors (6). According to the Chinese Society of Clinical Oncology (CSCO) Melanoma Diagnosis and Treatment Guidelines (2025), combined dabrafenib and trametinib, as well as its combination with immune checkpoint inhibitors such as pembrolizumab and toripalimab, are recommended as therapeutic options for patients with metastatic cutaneous melanoma. With the increasing combined application of targeted therapy and immunotherapy, the incidence of their ocular adverse reactions has increased correspondingly, mainly including mild lesions such as conjunctivitis and keratitis, while severe panuveitis, especially VKH syndrome-like uveitis, is extremely rare (7–9).

To date, no cases of bilateral VKH-like uveitis associated with the combined treatment of DabTram and cadonilimab have been reported, and the relevant clinical data and diagnosis and treatment experience are extremely scarce. Therefore, this study reports a clinical case of bilateral VKH-like uveitis in a melanoma patient after treatment with DabTram and cadonilimab. We elaborate on its clinical manifestations, imaging features, laboratory examinations, and diagnosis and treatment process in detail, and conduct a discussion in combination with relevant literature. The aim is to improve clinicians’ ability to identify such rare adverse reactions and provide a reference for optimizing the management of adverse reactions during anti-tumor therapy.

## 2 Case presentation

A 47-year-old female patient initially presented with an inguinal mass four months before admission and received surgical resection of the lesion at our hospital. Postoperative pathology confirmed cutaneous melanoma with regional inguinal lymph node metastasis. Subsequent inguinal lymph node dissection was performed for further staging evaluation. Immunohistochemistry showed tumor cells were positive for Vimentin, S100, HMB45 and Melan A, focally weakly positive for CK, negative for P40, with a Ki-67 proliferation index of 20%. Molecular testing identified a BRAF V600E mutation, and further systemic workup confirmed concurrent hepatic metastasis. Accordingly, oncology department initiated first-line combined antitumor therapy: oral dabrafenib 150 mg once daily plus trametinib 2 mg once daily, combined with intravenous cadonilimab 500 mg administered every 3 weeks. Following 4 months of consistent combined treatment, the patient spontaneously developed bilateral ocular redness, pain, and decreased visual acuity without obvious predisposing causes. These ocular symptoms persisted for 2 weeks, prompting her admission to our hospital on 9 December 2024. She denied photophobia, lacrimation, oral ulcers, tinnitus, alopecia, morning stiffness, or lumbago. She had no history of hypertension, diabetes mellitus, coronary artery disease, prior ocular disorders, eye surgery, or ocular trauma. No relevant familial or genetic predisposition was documented.

**FIGURE 1:**
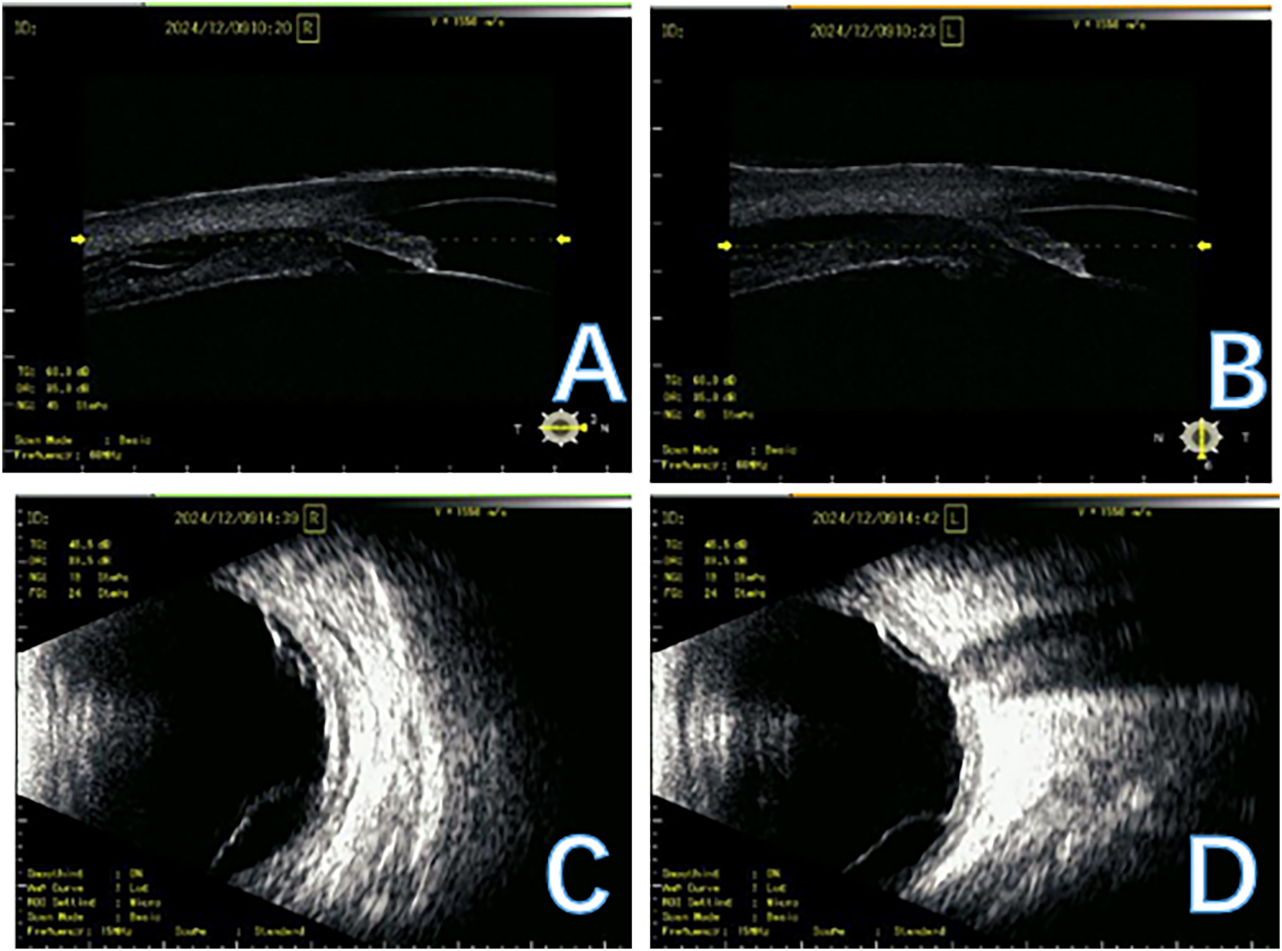
Ultrasound biomicroscopy (UBM) demonstrated scattered punctate echoes in the anterior chamber and anechoic spaces between the sclera and ciliary body in both eyes **(A,B)**. Ocular B-scan ultrasonography showed uniform and smooth band-like echoes in the peripheral parts of both eyeballs, with multiple arc-shaped anechoic spaces between the eyeball wall and sclera **(C,D)**.

**FIGURE 2:**
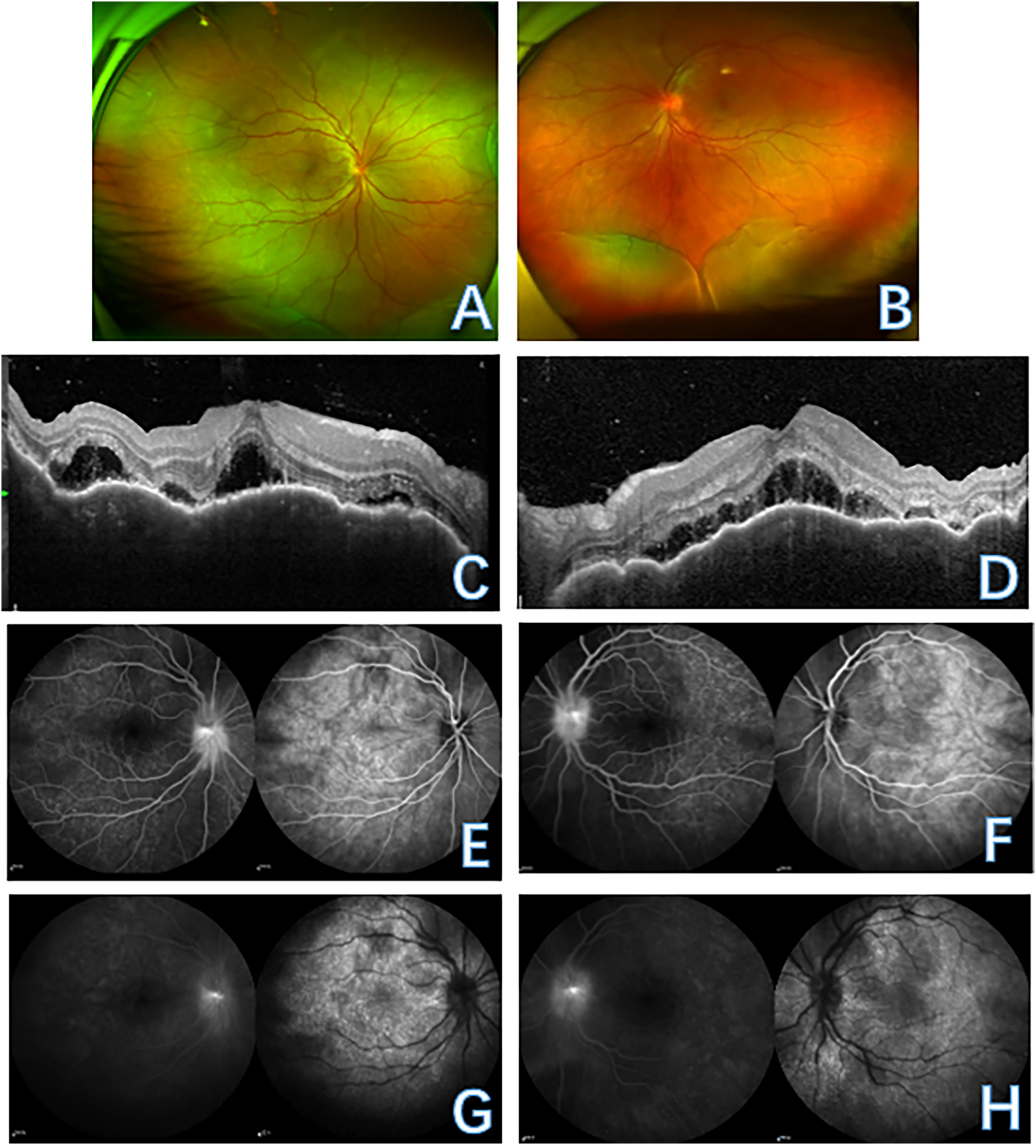
Scanning laser ophthalmoscopy (SLO) revealed edema of the optic disk in both eyes, peripheral retina exudative bulge detachment **(A,B)**. Optical coherence tomography (OCT) revealed serous retinal detachment and choroidal folds in both eyes **(C,D)**. Fundus fluorescein angiography (FFA) and indocyanine green angiography (ICGA) showed multiple pinprick hyperfluorescence in the posterior pole of both eyes and optic disk fluorescence leakage with blurred margins, scattered hypofluorescent dark spots were observed in the posterior pole and midperiphery **(E–H)**.

**FIGURE 3:**
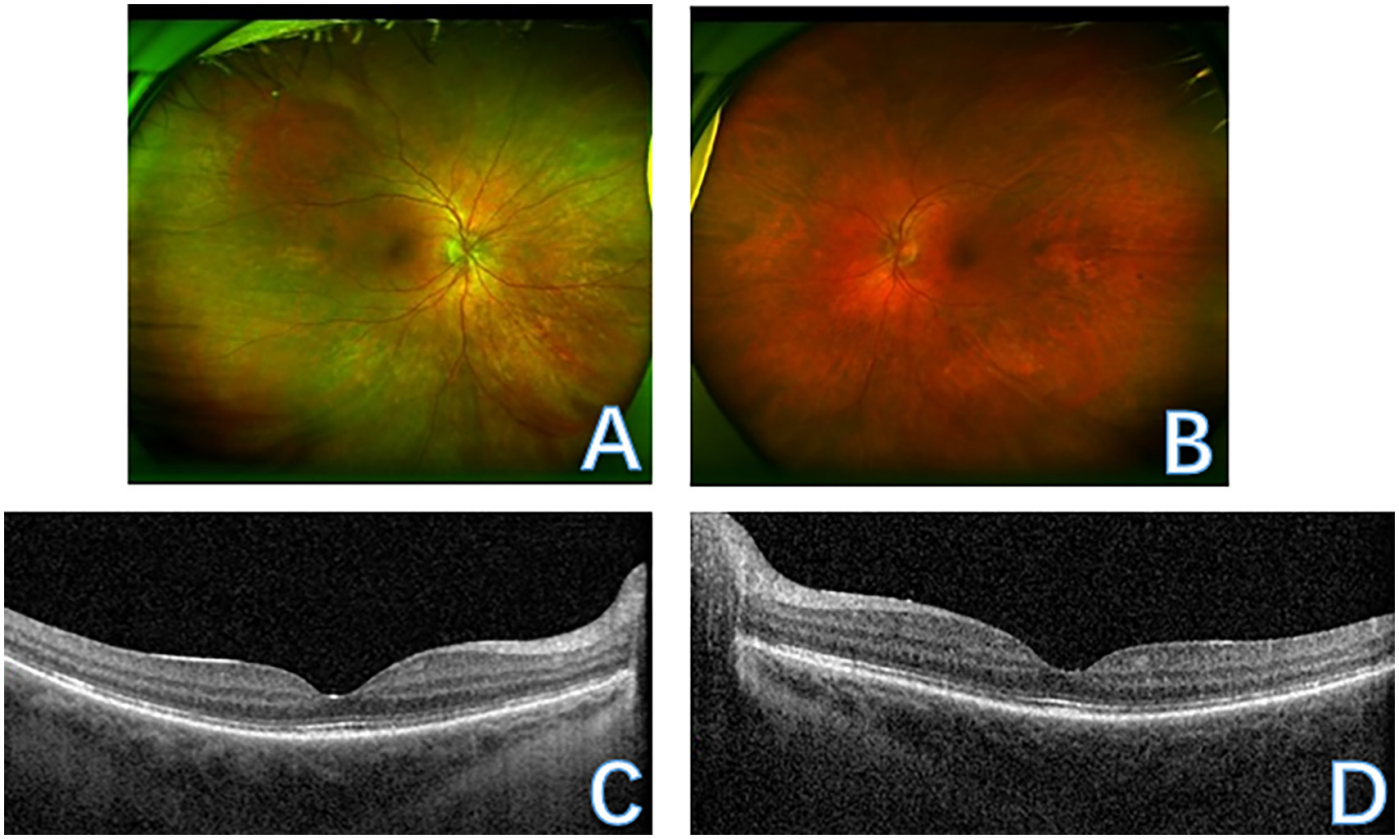
Scanning laser ophthalmoscopy (SLO) demonstrated a sunset-glow fundus appearance in both eyes **(A,B)**. Optical coherence tomography (OCT) showed a normal macular morphology with clear and continuous layers of the retinal structure in both eyes **(C,D)**.

Ophthalmic examinations showed that the best-corrected visual acuity (BCVA) was 20/200 in the right eye and 20/100 in the left eye, as measured with the Snellen visual acuity chart. The intraocular pressure (IOP) was 14 mmHg in the right eye and 13 mmHg in the left eye (1 mmHg = 0.133 kPa). Anterior segment examination of both eyes revealed dust-like keratic precipitates, positive aqueous cells (3+) and mild lens opacification. A small number of inflammatory cells were observed in the mid and posterior vitreous cavity. Ultrasound biomicroscopy (UBM) demonstrated scattered punctate echoes in the anterior chamber and anechoic spaces between the sclera and ciliary body in both eyes (Figures 1A, B). Ocular B-scan ultrasonography showed uniform and smooth band-like echoes in the peripheral parts of both eyeballs, with multiple arc-shaped anechoic spaces between the eyeball wall and sclera (Figures 1C, D). Scanning laser ophthalmoscopy (SLO) revealed multiple exudative retinal detachments involving the entire retina and optic disk edema (Figures 2A, B). Optical coherence tomography (OCT) revealed multifocal serous retinal detachments in the macular area of both eyes, with punctate moderate-to-strong reflective signals in the detachment cavities and vitreous cavity, wavy changes in the retinal pigment epithelium (RPE) layer, and diffuse choroidal thickening (Figures 2C,D). Combined fundus fluorescein angiography (FFA) and indocyanine green angiography (ICGA) showed multiple pinprick hyperfluorescences in the posterior pole of both eyes and optic disk fluorescence leakage with blurred margins in the early phase of FFA; scattered hypofluorescent dark spots were observed in the posterior pole and midperiphery on ICGA. In the late phase of FFA, the pinprick hyperfluorescences in both eyes showed further leakage and fusion, with persistent and aggravated optic disk leakage presenting as diffuse strong fluorescence with ill-defined margins; diffuse strong fluorescence was also noted on ICGA (Figures 2E–H). Laboratory examinations showed positive human leukocyte antigen B27 (HLA-B27) and an elevated erythrocyte sedimentation rate (ESR) of 57 mm/h (positive). Antinuclear antibody (ANA), ANA profile, Toxoplasmosis, Others, Rubella, Cytomegalovirus, and Herpes (TORCH) panel, HIV antibody, treponema pallidum antibody, hepatitis B surface antigen, high-sensitivity C-reactive protein, anticardiolipin antibody, antistreptolysin O antibody, rheumatoid factor and chest computed tomography (CT) were all normal. HLA-DRB1 allele detection revealed HLA-DRB109:01 and HLA-DRB111:01. Given that the patient developed uveitis 4 months after initiating targeted therapy, VKH-like uveitis secondary to DabTram and cadonilimab therapy was considered. Given the need for continued anti-tumor treatment, the DabTram therapy was discontinued, while cadonilimab immunotherapy was continued. The patient was treated with oral prednisone acetate 40 mg daily, with a tapering regimen of 10 mg reduction every 2 weeks until a maintenance dose of 10 mg daily was achieved at 6 weeks. Topical ophthalmic medications included tobramycin and dexamethasone eye drops, pranoprofen eye drops and compound tropicamide eye drops. Two weeks after discontinuation of dabrafenib/trametinib and initiation of steroid therapy, the patient’s symptoms and visual acuity improved. Four months later, she had no obvious ocular discomfort, and the BCVA of both eyes improved to 20/25. SLO and OCT showed complete resolution of multifocal serous retinal detachments (Figures 3A–D). During the 1-year follow-up after discontinuation of dabrafenib/trametinib and continuous cadonilimab treatment, no visual deterioration or new ocular symptoms were noted, and regular imaging confirmed stable hepatic metastases without tumor progression or newly developed metastatic lesions. Meanwhile, oral corticosteroid therapy was discontinued. A timeline of key clinical events is provided in Supplementary material.

## 3 Discussion

Mitogen-activated protein kinase /extracellular signal-regulated kinase (ERK) is a highly conserved serine/threonine protein kinase in eukaryotes that plays a pivotal role in cell proliferation and apoptosis. It also serves as a regulatory hub for various signaling pathways involved in angiogenesis and is closely associated with tumor growth and metastasis (10). BRAF is one of the most important proto-oncogenes in humans; its mutation leads to constitutive activation of the MAPK signaling pathway, thereby driving the proliferation, growth and differentiation of tumor cells (11). Consequently, both MAPK-ERK kinase (MEK) and BRAF have become key therapeutic targets for tumor therapy in recent years. The combination of the BRAFi and the MEKi is currently the only approved combination regimen in China for the adjuvant treatment of patients with completely resected stage III melanoma harboring the BRAF V600 mutation (12). Cadonilimab is a bispecific antibody targeting human PD-1 and CTLA-4. It blocks the interactions of PD-1 and CTLA-4 with their respective ligands, thereby abrogating the immunosuppressive effects of the PD-1 and CTLA-4 signaling pathways, promoting the activation of tumor-specific T-cell immunity, preventing immune escape of tumor cells, and ultimately exerting an anti-tumor effect (8). Although the widespread application of the aforementioned drugs has improved the therapeutic landscape for some cancer patients, their potential to induce relevant ocular adverse reactions cannot be ignored. A summary of previously reported cases of VKH-like uveitis associated with DabTram and cadonilimab is presented in Table 1. The patient in this case had normal visual acuity before anti-tumor therapy and no previous history of ocular diseases or surgery. She developed VKH-like syndrome symptoms after administration of DabTram and a PD-1 inhibitor. Laboratory tests for ANA, TORCH, HBsAg, HIV and other markers were unremarkable. Her symptoms improved after the discontinuation of DabTram alone, but follow-up FFA still revealed fluorescent leakage of retinal blood vessels and persistent depigmentation of facial skin. However, it remains unclear whether the uveitis was induced by dabrafenib plus trametinib or by cadonilimab. Given the clear temporal association with drug administration, a final diagnosis of drug-related VKH-like uveitis was made.

**TABLE 1:** Summary of published case reports of Vogt-Koyanagi-Harada (VKH)-like uveitis secondary to dabrafenib and trametinib or cadonilimab.

References	Age/gender	Primary tumor characteristics	Antitumor treatment regimen	Genetic / HLA status	Management of VKH-like uveitis	Ophthalmic outcome	Visual acuity at presentation	Final BCVA	Follow-up duration
Nakano et al. (13)	36/M	MCM	D+T → I+N	HLA- DRB1*03:01, 12:02	Discontinued D/T + corticosteroid + adalimumab	Visual improvement	HM OD, HM OS	1.0 OD, 0.9 OS	5 months
Gunaratnam et al. (16)	49/F	Stage → MCM	D+T	NA	Discontinued D/T + systemic corticosteroids	Complete resolution	6/9 OD, 6/5 OS	6/6 OD, 6/6 OS	4 months
Ucan Gunduz et al. (9)	49/F	Recurrent MCM	D+T	NA	Discontinued D/T + systemic corticosteroids	Complete resolution	0.5 OD, 0.3 OS	1.0 OD, 1.0 OS	4 years
Sada et al. (25)	39/M	Malignant melanoma	D+T	DRB1*04:05	Oral corticosteroid	Visual improvement	NA	NA	NA
Sada et al. (25)	45/F	Vulva malignant melanoma	D+T	DRB1*04:06	discontinued D/T + oral corticosteroid + topical	Visual improvement	NA	NA	NA
Madoe et al. (26)	49/M	MM	N → D+T	NA	Discontinued D/T + systemic corticosteroids	Complete resolution	20/63 OD, 20/20 OS	20/20 OD, 20/20 OS	13 months
Amagai et al. (14)	35/F	Stage → advanced melanoma	D+T → N	HLA-DRB1*04:05, 08:03	IV steroids	Vision restored	NA	NA	NA
Amagai et al. (14)	73/M	Stage → advanced melanoma	D+T → N	HLA-DRB1*04:05, 04:10	IV steroids	Vision restored	NA	NA	NA
Amagai et al. (14)	26/F	Stage → advanced melanoma	D+T	HLA-DRB1*04:05, 15:01	IV steroids	Vision restored	NA	NA	NA
Bellanca et al. (27)	51/F	CM	D+T	NA	Discontinued D/T + systemic corticosteroids	Complete resolution	HM OD, HM OS	20/20 OD, 20/20 OS	10 months
Kaymak et al. (28)	54/F	CM	D+T	NA	Discontinued D	Complete resolution	0.3 OD, 0.15 OS	NA	NA
Brambati et al. (29)	53/M	MCM	D+T	NA	Discontinued D/T + IV steroids + oral corticosteroid	Complete resolution	HM OD, 20/400 OS	20/25 OD, 20/25 OS	6 months
Woltsche et al. (30)	59/M	MCM	D+T	NA	Discontinued D/T; subconjunctival corticosteroids + intravitreal corticosteroids and bevacizumab	Retinal improvement	20/20 OD, 20/20 OS	20/20 OD, 20/20 OS	12 months
Rueda-Rueda et al. (31)	39/F	MM	D+T	NA	Discontinued D/T + IV steroids + oral corticosteroid + topical	Retinal improvement	0.2 OD, 0.1 OS	1.0 OD, 1.0 OS	6 months
Fujimura et al. (32)	73/M	Stage IIIC superficial spreading melanoma	N → D+T	HLA-DRB1*04:05	Discontinued D/T + IV steroids + oral corticosteroid	Visual improvement	NA	NA	NA
Fujimura et al. (32)	35/F	Advanced melanoma	N → D+T → N	HLA-DRB1*04:05	IV steroids	Visual improvement	NA	NA	NA
Draganova et al. (33)	55/F	MCM	D+T	NA	Discontinued D/T + topical corticosteroids	Visual and retinal improvement	HM OD, CF OS	20/25 OD, 20/25 OS	1 month

CF, counting fingers; CM, cutaneous melanoma; D, dabrafenib; F, female; HM, hand motion; I, ipilimumab; IV, intravenous; M, male; MCM, metastatic cutaneous melanoma; MM, metastatic melanoma; N, nivolumab; NA, not available; OD, oculus dexter; OS, oculus sinister; T, trametinib.

The patient developed symptoms 4 months after drug initiation, which is consistent with the time of onset of drug adverse reactions reported in previous studies (7, 13, 14). However, close monitoring is still required during clinical medication to avoid missed diagnosis due to insidious early symptoms. Four months after the discontinuation of DabTram, follow-up FFA still showed bilateral retinal vascular fluorescent leakage, and localized facial skin depigmentation persisted. In contrast to the nivolumab-associated VKH-like uveitis reported by Imai et al. (15). which completely resolved despite continued nivolumab, our patient showed incomplete resolution with persistent FFA leakage and facial depigmentation. We propose the following explanations for this difference. First, cadonilimab is a PD-1/CTLA-4 bispecific antibody. Unlike nivolumab which blocks only PD-1, cadonilimab simultaneously removes two major immune checkpoints. CTLA-4 is crucial for maintaining peripheral tolerance; its blockade leads to broader and more sustained T-cell activation against melanocyte antigens, potentially causing longer-lasting tissue damage that is less reversible. Second, the bispecific design may disrupt ocular immune privilege more profoundly. Dual blockade can more effectively suppress local regulatory circuits, allowing effector T-cells to persist within the uvea and skin even after acute inflammation subsides. Third, genetic background likely plays a modifying role. Our patient carried HLA-DRB1*09:01 and *11:01, alleles not previously linked to VKH, whereas Imai’s patient was HLA-DR4-positive. We hypothesize that these different HLA alleles may influence T-cell persistence or tissue recovery, interacting with the stronger immune activation from dual blockade to produce incomplete resolution. We did not resume dabrafenib/trametinib in this patient. Although Gunaratnam et al. (16) demonstrated that dose-adjusted reintroduction of DabTram may be feasible in selected patients, we refrained from rechallenge for the following reasons: the patient had severe bilateral panuveitis at onset, multimodal imaging (FFA and OCT) showed persistent inflammatory leakage even after clinical improvement, and recurrence of VKH-like uveitis would carry a substantial risk of irreversible visual loss. Moreover, the patient’s metastatic melanoma remained stable on cadonilimab monotherapy, so there was no urgent oncologic indication to restart targeted therapy. Therefore, cadonilimab was continued as the sole anti-tumor treatment, and the decision not to reintroduce DabTram was made jointly with the oncology team after careful risk-benefit assessment. Long-term effects on visual function and changes in retinal structure require continuous follow-up.

The pathogenesis of DabTram and PD-1 inhibitor-associated VKH-like uveitis remains unclear to date, but it is speculated to be related to the following factors: first, melanoma and uveal melanocytes share identical surface proteins: BRAF inhibitors can induce the generation of cytotoxic T lymphocytes targeting melanocytes, which in turn leads to melanoma cell apoptosis (17). Since uveal and cutaneous melanocytes express the same surface proteins, this cross-reactivity results in damage to both uveal and cutaneous melanocytes (18); second, excessive activation of the autoimmune system: Uncontrolled T-cell activation following immune checkpoint inhibition causes the body’s normal cells and healthy tissues to be attacked by the immune system, thereby triggering a series of immune-related inflammatory responses and leading to VKH-like uveitis (19); third, disruption of the ocular immune privilege: Corneal epithelial cells and RPE cells express the ligand for PD-1 (PD-L1) on their surface. The interaction between PD-1 and PD-L1 generates inhibitory signals that suppress T-cell signal transduction and prevent immune cell activation, thus avoiding immune responses against host cells—this is one of the key mechanisms underlying ocular immune privilege (20, 21). Blockade of the PD-1 pathway disrupts ocular immune privilege, leading to immune cell activation, subsequent RPE cell damage and breakdown of the blood-retinal barrier; fourth, presence of VKH susceptibility genes: Amagai et al. (14) performed HLA genotyping in five melanoma patients with DabTram-associated VKH-like uveitis and found all were positive for HLA-DRB104. Takeuchi et al. (22) conducted genetic testing in four cancer patients with PD-1 inhibitor-associated VKH-like uveitis and reported universal positivity for HLA-DRB104:05. The patient in this case was positive for HLA-DRB109:01 and HLA-DRB111:01, and no literature has reported an association between these genotypes and VKH syndrome. However, we hypothesize that VKH-like uveitis induced by such targeted drugs may share a similar pathogenesis with classic VKH syndrome. The presence of these genes confers a higher risk of relevant antigen exposure compared with patients without these genes, making them more prone to developing VKH-like uveitis during targeted drug therapy. Available evidence remains limited and mainly comes from small observational studies. Further large-sample and in-depth studies are still required to clarify the relationship between these susceptibility genes and disease onset risk.

There is no clear and unified therapeutic regimen for drug-related uveitis to date. Mettler et al. (23) recommended that BRAFi should be discontinued first in cases with anterior segment involvement, MEKi for posterior segment involvement, and both BRAFi and MEKi for intermediate uveitis or panuveitis. If adverse reactions resolve, targeted therapy may be restarted under close monitoring based on the severity of previous symptoms and the necessity of treatment. Dow et al. (24) suggested that for PD-1 inhibitor-associated ocular adverse reactions, grading can be performed according to the Common Terminology Criteria for Adverse Events in the Biologic Agent Adverse Reaction Guidelines, and an individualized treatment plan should be formulated based on the adverse reaction grade and the opinions of the oncology department. The patient in this case presented with bilateral panuveitis. After a joint assessment of the patient’s condition with oncologists, DabTram was discontinued, and anti-inflammatory eye drops plus oral prednisone acetate at a dose of 1 mg⋅kg^–1^⋅d^–1^ were administered. The patient’s visual function improved 2 weeks later, and a sequential tapering of prednisone acetate was initiated. Four months later, the patient’s BCVA improved to 0.8, but FFA still indicated inflammatory changes in the retinal blood vessels. This finding is in contrast to the case reported by Ucan Gunduz et al. (9), in which inflammation resolved completely following drug cessation and corticosteroid treatment. Such a difference may be attributable to the continued administration of cadonilimab in the present case, as well as the distinct HLA profile. This indicates that clinicians should conduct intensified long-term follow-up for ocular and cutaneous manifestations in patients in whom immune checkpoint inhibitors are maintained.

In summary, this case is the first report of Vogt-Koyanagi-Harada-like uveitis associated with the combination of dabrafenib/trametinib and cadonilimab. It shows similarities in clinical manifestations and treatment response to previously reported cases induced by similar classes of drugs, but exhibits uniqueness in drug combination, HLA genotype, and persistent inflammation status. These similarities and differences provide new clinical evidence for understanding the impact of different anti-tumor drug combinations on the occurrence and outcome of uveitis, and also suggest that genetic background may play a modifying role in drug-associated VKH-like inflammation. At the same time, effective management of patient compliance and multidisciplinary collaboration are essential to reduce the risk of inflammatory progression and improve patients’ visual prognosis.

## Data Availability

The original contributions presented in this study are included in the article/Supplementary material, further inquiries can be directed to the corresponding author.

## Supplementary material

Supplementary Figure 1 Timeline of the patient’s diagnosis and treatment process.
