# Supplementary figures and images for: Case Report: Vogt-Koyanagi-Harada-like uveitis associated with combined dabrafenib/trametinib and cadonilimab therapy for cutaneous melanoma
Source: Front Med (Lausanne). 2026 Jun 24;13:1804008. doi: 10.3389/fmed.2026.1804008 (PMC13342179; doi:10.3389/fmed.2026.1804008)

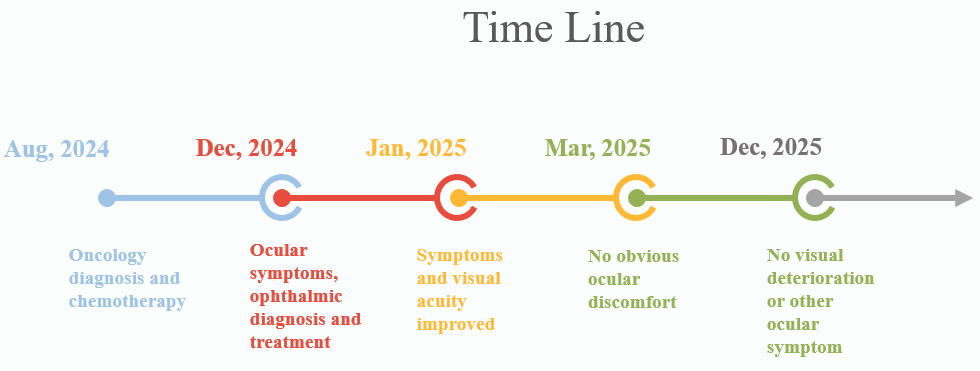

Supplement: Supplementary Figure 1 — Timeline of the patient’s diagnosis and treatment process. [file Image_1.TIF]
